# Clinical Diagnosis

**Published:** 1894-10

**Authors:** 


CLINICAL DIAGNOSIS. By Albert Abrams, M. D. (Heidelberg). Third edition. Revised, enlarged, and illustrated. New York: E. B. Treat, No. 5 Cooper Union, 1894. Price, $2.75.

This work is an octavo volume of 275 pages, giving the latest methods of diagnosis, especially in reference to heart, lung, and kidney troubles. There are, of course, many such books before the profession, but this one, as it gives the latest methods, is the best. The best method of familiarizing the ear with the valvular sounds in health and in disease is given. The latest methods for testing the urine for abnormal products and the latest views upon the great question of Bright's disease may here be found briefly yet clearly given. The original design of the book was as an index to more pretentious works on the subject of diagnosis. This design has not been lost sight of in this third edition. Very elaborate tables upon heart sounds, upon urinary tests, upon the different kinds of microbes are given to enlighten the earnest seeker for knowledge, and a chapter upon the differential diagnosis of the various forms of insanity is added as an appendix.

